# A Case of Impacted Gall-Stone

**Published:** 1910-05-21

**Authors:** 


### SURGERY.

### A CASE OF IMPACTED GALL-STONE.

Cases in which a gall-stone leaves the gall-bladder through an unnatural opening and makes its way into some part of the alimentary tract are sufficiently rare to be interesting, although, of course, such a condition is recognised in the text-books of surgery as a cause of acute intestinal obstruction. The following case is worthy of record as presenting some unusual features.

The patient, a woman, aged 51, was suddenly seized with acute pain in the abdomen, described by herself as being "of a griping nature and situated round about the navel." In other words, it was in the nature of a paroxysmal colic. The only other thing she could say about it was that it was "rather like a liver attack." For thirty years she had been a victim to these "liver attacks." They were characterised by pain and tenderness over the region of the liver, but had never been associated with jaundice or any change in the colour of her motions. She was always constipated, and this was more obstinate during the attacks. At these times she was given medicine by her doctor and the pain and tenderness subsided.

### Course of the Case.

But in certain respects her present illness did not conform entirely to the type of her past attacks. The griping pain, for instance, was a new feature, and soon after its onset she began to vomit, which she hardly ever had done before. Further, she took her customary medicine, but this failed to give her any relief. On the contrary, the pain became steadily worse, and she continued to vomit brown matter which was not fæcal. Neither flatus nor fæces were passed. After forty-eight hours a hypodermic injection of morphia was given her by her doctor and her pain disappeared, but the vomiting continued. She then came under the writer's observation. She was an edentulous woman, but did not otherwise appear older than her years. She was well covered, and did not present the appearance of a patient with malignant disease. At the same time she was obviously acutely ill. Her temperature was subnormal and her pulse small and rapid (140 to the minute). Her pupils were pinpoint and she had no pain: this was, of course, the result of the morphia; but she continued to vomit, and the vomit was now definitely stercoraceous. The abdomen was somewhat uniformly distended: but, she said, not more so than usual. It was resonant on percussion all over, but not tympanitic. No mass was felt. Palpation on the left side, and particularly in the left iliac fossa, induced vomiting immediately. There was no evidence of a hernia at any of the ordinary orifices. Rectal examination revealed no abnormality. There were no piles, the sphincter was not relaxed, and the rectum not ballooned. No growth was felt.

It was obvious that the patient was suffering from acute intestinal obstruction, but the actual cause was not evident.

### The Findings at Operation.

An operation was immediately performed. An incision was made through the right rectus just to the right of the middle line, four inches long, with its centre below the umbilicus. Distended and injected small intestine immediately presented. The cæcum was sought for, and found collapsed. The appendix was normal. On tracing the ileum back from the ileo-cæcal valve, a hard body was found impacted in it about two feet from the valve. This was obviously the cause of the obstruction, since the intestine proximal to it was distended, while that on the distal side was collapsed. An incision was made in the gut and the body removed. It proved to be a large gall-stone, roughly the size of a pigeon's egg, with a concave facet on its smaller end. It had occupied the fundus of the gall-bladder. The wound in the intestine was sewn up with Lembert sutures. And now comes the unusual feature of the case. The pelvis was explored to eliminate any other possible cause of obstruction, and in the left half of the pelvis a stony hard mass, the size of a turkey's egg, with an irregular surface, was felt. This was bound down by adhesions. These were freed, and on bringing the mass up to the surface it was found to be connected with the left Fallopian tube. It was rapidly removed after ligature of the tube, and turned out to be a calcified dermoid cyst of the left ovary. A hand was then passed up to the gall-bladder region, and the parts were found to be matted into an unrecognisable mass by dense adhesions. These were left severely alone. The wound was rapidly sewn up. The patient was extremely collapsed during the operation, and strychnine ♏ x. were injected. She never really rallied, and died of shock three hours after the operation. An autopsy was held and the exact state of affairs in the region of the gall-bladder was explored. The fundus of the gall-bladder had become adherent to the first part of the duodenum and the gall-stone had worked its way through, forming an artificial cholecystenterostomy, which was firmly shut off by adhesions. A second round gall-stone, smaller than the one removed from the gut, was found in the cystic duct. This had not begun to travel. It is commonly said in the text-books that if the gall-bladder communicates in this manner with any part of the intestine it usually does so with the hepatic flexure of the colon. But the writer has seen three such cases in the last twelve months, and in all the fistulous communication occurred into the duodenum; and in all acute intestinal obstruction followed from impaction at the lower end of the ileum. Why at this point? It is not likely that the stone meets the ileo-cæcal valve and then travels back by antiperistalsis. Is it possible that the intestine is slightly constricted at this point by a Meckel's diverticulum, which had left no microscopical traces? The question is one which anatomists might investigate.

